# Phase change dispersion of plasmonic nano-objects

**DOI:** 10.1038/srep12665

**Published:** 2015-07-29

**Authors:** Xie Zeng, Haifeng Hu, Yongkang Gao, Dengxin Ji, Nan Zhang, Haomin Song, Kai Liu, Suhua Jiang, Qiaoqiang Gan

**Affiliations:** 1 Material Science Department, Fudan University, Shanghai, China 200433; 2 Department of Electrical Engineering, The State University of New York at Buffalo, Buffalo, NY 14260; 3 College of Information Science and Engineering, Northeastern University, Shenyang 110819, China; 4 Alcatel-Lucent Bell Labs, Murray Hill, NJ 07974.

## Abstract

Phase is an inherent and important feature for coherent processes, which, unfortunately, has not been completely understood for surface plasmon polariton (SPP) and matter interactions. Here we propose a practical approach to extract the phase change dispersion during the interaction between free-space light, SPPs and nanogroove/slit based on far-field information only. Numerical simulation and experimental validation were both presented using nanoslit-groove plasmonic interferometers, agreeing well with theoretical near-field analysis. This approach is generally feasible to extract the intrinsic phase dispersion of other plasmonic nanostructures and can reveal more fundamental features of SPP-matter interactions.

The investigation on light interaction with individual slits has a long history in physical optics, resulting in many fundamental progresses (e.g. wave-particle duality). In recent years, there has been considerable growth in plasmonics research due to their subwavelength confinement of electromagnetic (EM) waves^1^. To better understand surface plasmon polariton (SPP) mediated interactions between optical nano-objects at metallodielectric interfaces, researchers have performed numerous theoretical and experimental studies of the physics of simple nanostructures, e.g., single or double slits^2^,^3^,^4^, grooves^5^,^6^,^7^,^8^,^9^, or apertures^10^,^11^,^12^,^13^. But even for the simplest single slit-groove structure, some initial debate arose regarding the physical mechanisms underlying these interactions^14^,^15^,^16^,^17^. Therefore, there are still many open questions regarding fundamental understanding on SPP waves.

Phase is an inherent and important feature for coherent processes, particularly for EM waves (e.g. high resolution imaging^18^ , quantum optics^19^, etc.). As reported in a double slit SPP interference experiment^20^, the phase information is essential to interpret the spatial interference pattern of SPP waves, reflecting the plasmonic wave-particle duality of quantum mechanics^21^. In recent years, we have witnessed a rapid growth of plasmonics investigation involving the study of the intrinsic properties of SPP waves (including phase information^17^,^22^,^23^,^24^) and their interaction with matter at nanoscales^25^. For instance, during the interaction between light and subwavelength slits/grooves, those slits/grooves were considered as oscillating dipole sources and the excited SPPs were claimed to have a π intrinsic phase shift introduced by the process of charge accumulation at these simplified dipoles^23^,^24^. However, these works neglected the effect of nanosturctures’ shapes, and therefore, cannot reveal the intrinsic phase shift from these nanostructures accurately. Especially, the understanding on the intrinsic phase shift changes versus light wavelength, i.e. the dispersion of the intrinsic phase shift, is still missing. To our knowledge, no work explored the relationship between intrinsic phase shift versus wavelength, i.e. the intrinsic phase change dispersion. Particularly, this information will be very useful for recently emerging hologram metasurfaces^26^. On the other hand, previous works are either theoretical calculation based on simplified assumption (i.e., a single groove was considered as a dipole, see Ref. [24]), or semi-analytical method (e.g. Ref. [22]) which requires near-field information that is very difficult to obtain from experiments directly (i.e., the total field at the surface including surface plasmon modes and scattering components). Therefore, the beautiful theoretical work presented by ref. [22] has not been validated experimentally.

In this manuscript, we propose a simple far-field method based on plasmonic interferometers to extract the intrinsic phase shift at different wavelengths from both simulation and experiment. This method contains two steps: 1) interference fringe data acquisition; 2) peak/valley recognition and phase extraction. Experimentally, we employ a tilted slit-groove plasmonic interferometer to acquire the spatial interference fringe versus slit-groove distance. At each wavelength, transmission image of the tilted slit-groove interferometer is captured on a CCD camera through a regular optical microscope, and a subsequent data processing program can extract the intrinsic phase shift instantly. Therefore, this method is quite practical and convenient for experimental realization. It should be noted that this method does not require any detailed shape information on the nanogroove/slit. In other words, the phase dispersion for any groove-like pattern (e.g. single or multiple grooves, sphere shaped shallow patterns, triangles or even stars, etc.) can be extracted as long as it can generate plasmonic interference signal.

This article will be organized as follows: We will first explain the physical mechanism of the phase mismatch observed in previous literature and propose a practical method to extract the phase dispersion. Then, we will employ previously reported microscopic theory to plot the intrinsic phase dispersion and compare it with our extracted data from numerical modeling. Finally, experimental results will be presented to validate the proposed method as well as the microscopic theory.

**Phase mismatch**

To demonstrate the intrinsic phase dispersion during the light-SPP-nano-object interaction, here we employ a slit-groove structure as shown in Fig. 1(a) to obtain spectral interference patterns containing phase information of SPP waves. The slit width, groove width and depth are represented by *w*_*1*_*, w*_*2*_ and *d*, respectively. When a transverse magnetic (TM) polarized incident light impinges on the nanostructures, the groove couples the free-space light into SPPs on the metal-dielectric (i.e., Ag/Air) interface, which will propagate to the slit and interfere with the directly transmitted light through the slit in the far field. Therefore, the transmitted intensity through the slit, *I*_*t*_, can be described as


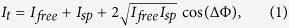


where *I*_*free*_ and *I*_*sp*_ are intensities of directly-transmitted free-space light and SPPs, respectively. The interference phase term can then be expressed as^14^


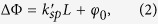


where 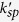 represents the real part of the propagation constant of SPPs, 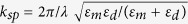 . Here *λ* is the incident wavelength; *ε*_*m*_, *ε*_*d*_ are permittivities of metal and dielectric materials on the interface, respectively. *L* is the propagation distance of SPPs between the slit and groove, and *φ*_*0*_ is an intrinsic phase shift occurred in the SPP excitation and rescattering processes at the nanogroove and nanoslit^4^,^14^,^17^. In previous reports^27^,^28^,^29^, *φ*_*0*_ was considered as a constant for all wavelengths, which resulted in a phase mismatch between theoretical and practical interference fringes. To demonstrate this mismatch, we first modeled the spectral interference pattern of a slit-groove interferometer with *L* = 10 μm, *w*_*1*_ = 130 nm, *w*_*2*_ = 230 nm and *d* = 70 nm. As shown in Fig. 1(b), the black spectral interference fringe is numerically modeled by finite difference time domain (FDTD) method (see Section I in the Supplementary Information). The blue curve is the analytical interference pattern governed by the cos(•) term in Eq.(1) by considering *φ*_*0*_ as a constant at all wavelengths. In this modeling, the constant value of *φ*_*0*_ is selected to make the two interference fringes match at the first peak (i.e. ~520 nm). An obvious mismatch of the peak/valley positions between them can be observed at longer wavelengths. Therefore, the dispersion of *φ*_*0*_(λ) is not negligible, which will be extracted in this work.

**Proposed approach to extract the intrinsic phase φ0(λ) from far-field information**

According to Eqs. (1) and (2), since ΔΦ varies linearly with *L* at a fixed wavelength, interference fringes should exhibit a constant period versus *L*, which is verified by the simulation results shown in Fig. 2(a). In this modeling, we modeled the *L*-dependent interference fringes at the wavelength of 520 nm, 620 nm, 720 nm and 820 nm, respectively, showing constant periods which agree well with theoretical predictions. The interference amplitude decreases slightly with increasing *L* due to the propagation loss of SPP modes. By extracting the interference peaks and valleys, ΔΦ at each wavelength can be fitted as a straight line shown in Fig. 2(b). The intrinsic phase shift *φ*_*0*_(λ) at each wavelength λ is the intercept of each fitted straight line on ΔΦ axis [i.e. *L* = 0, see the inset of Fig. 2(b)]. As shown in Fig. 2(c), the extracted *φ*_*0*_(λ) are plotted by blue diamonds in [0,2π) regime (with the wavelength resolution of 4 nm), and the red dashed line represents its polynomial fitting. One can see that *φ*_*0*_(λ) decreases monotonically with increasing λ in the spectral regime from 520 nm to 820 nm. By substituting these dispersive phase change data into the analytical Eq. (1) to plot the spectral interference pattern as shown by the red dashed curve in Fig. 1(b), one can see that the numerically and analytically modeled interference fringes match very well with each other.

**Microscopic understanding of Intrinsic Phase Dispersion**

To explore the physical mechanism of intrinsic phase dispersion of plasmonic nano-objects at metallodielectric interfaces, we now employ a microscopic model^22^ to interpret the phase shift introduced in scattering processes in the slit-groove interferometer. According to previous literature, this model has been used to quantify the SPP generation efficiency^2^,^7^,^30^, its contribution to extraordinary optical transmission^13^, as well as to optimize the design of plasmonic nano-antennas for efficient SPP coupling^2^,^9^,^31^,^32^. In these reports, generation efficiencies of SPP modes were analyzed in details based on near-field information modeled by numerical simulation (i.e., the total field at the surface including surface plasmon modes and scattering components^2^,^22^), which, unfortunately, is challenging to be validated experimentally. In this section, we will employ this near-field method to analyze the phase factors of involved scattering processes, and compare them with the dispersive phase extracted by our far-field method.

Based on the unconjugate general form of mode orthogonality condition^33^, the complex amplitudes of SPP mode propagating on the top metal-dielectric interface can be calculated using the following equation^2^,^22^,^34^:


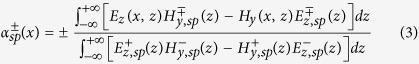


Here the total field {*H*_*y*_(*x,z*), *E*_*z*_(*x,z*)} can be simulated numerically using full-wave EM modeling, while the SPP field {*H*_*y,sp*_(*z*), *E*_*z,sp*_(*z*)} can be obtained analytically. The ‘+’ and ‘−’ are used to distinguish the forward- and backward-propagating SPP waves along x-axis, whose intensity and phase can be expressed by 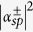
and 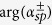, respectively. The nanoslit can be considered as a metal-insulator-metal (MIM) waveguide with a narrow width to support fundamental mode only. Therefore, the complex amplitudes of MIM fundamental modes, 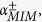
can also be calculated by properly modifying the subscripts and coordinates of Eq. (3)^34^. Details of theoretical derivations are listed in Section II of SI.

As illustrated in Fig. 3(a), four scattering processes are involved in the interaction between two interfering components. In the SPP coupling channel, three scattering processes contribute to the phase change in addition to the phase accumulation during the wave propagation: i.e., ① The incident free-space light is coupled into SPPs at the groove; ② SPPs arriving at the slit is scattered into the MIM fundamental mode propagating in the slit; and ④ the MIM fundamental mode is rescattered to free-space light at the output port of the slit. While for the direct-transmission channel, only two scattering processes are involved: i.e., ③ the incident free-space light is coupled into MIM fundamental mode at the slit input port; and ④ the MIM fundamental mode is scattered into free-space light at the output side of slit. Since the two channels share the scattering process ④, the intrinsic phase difference, *φ*_*0*_, should be contributed by scattering processes ① –③ only:


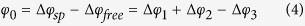


where Δ*φ*_*sp*_ and Δ*φ*_*free*_ represent phase shifts introduced by the nanostructure scattering in the SPP channel (see the red path in Fig. 3a) and direct-transmission channel (see the black path in Fig. 3a), respectively. Δ*φ*_1_, Δ*φ*_2_ and Δ*φ*_3_ correspond to the phase change in each scattering process, as described below:


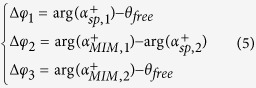


As shown in Fig. 3(a), 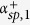 and 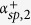
represent the complex amplitudes of forward-propagating SPP generated at the groove and arriving at the slit, respectively; 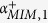 and 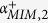 are complex amplitudes of MIM fundamental mode coupled from SPP and free-space light, respectively; *θ*_*free*_ is the phase of the incident plane wave at the metal surface. At each wavelength, all these parameters can be calculated by the near-field method described above. Fig. 3(b) shows the intrinsic phase dispersion contributed by scattering processes occurred at the groove and slit, i.e. Δ*φ*_1_ and (Δ*φ*_2_ − Δ*φ*_3_), respectively. It should be noted that processes ② and ③ were completely ignored in a previous theoretical work^23^, which is an inaccurate assumption as shown by the dispersion of modeled Δ*φ*_2_ (blue line) and Δ*φ*_3_ (green line) in the inset of Fig. 3(b). One can see that all these phases show a monotonous decrease in the studied spectral region, and the SPP excitation process at the groove plays a larger role in the intrinsic phase dispersion (i.e. Δ*φ*_1_  >  (Δ*φ*_2_ − Δ*φ*_3_)). By summing up Δ*φ*_1_ and (Δ*φ*_2_ − Δ*φ*_3_) in these two figures, the total intrinsic phase shift versus wavelength is drawn by black circles in Fig. 3(c), agreeing very well with the results extracted from far-field information (i.e., blue diamonds from Fig. 2(c)), demonstrating the feasibility of the proposed phase extraction approach. It should be noted that there is still a slight mismatch between the phase dispersion obtained by the near-field and far-field analyses, which should be attributed to the numerical simulation error introduced by the mesh size of FDTD modeling and the potential contribution of cylindrical waves^3^,^13^,^15^,^35^,^36^. Remarkably, our proposed phase extraction method relies on far-field information only, which can be easily validated using experiment, as will be discussed in the next section. In contrast, the microscopic theory is challenging since the total field at the surface (including SPP modes, radiative modes, etc.^2^,^22^) is required, which is difficult to be obtained conveniently from experiment.

**Experimental Validation**

Since the proposed procedure requires far-field optical information only (i.e., the distance-dependent interference patterns at different wavelengths), the phase change dispersion during the SPP-matter interaction can then be straightforwardly extracted from experiment. Here we fabricate tilted slit-groove plasmonic interferometers^37^,^38^ on Ag/Al/Ti (70 nm/200 nm/10 nm) metal films using focus ion beam (FIB) milling technique. Using this structure, a range of *L* can be covered in a single experiment, which is significantly simpler than previously reported experiments using a series of structures with finely tuned distance *L* (e.g.^14^,^17^,^39^). Since the FIB etching rate for Al is much smaller than that for Ag, the Al layer can function as a stopping layer for ion beam milling to obtain better shape and bottom roughness control^31^ (see Fig. S2 in SI for more information). As shown in Fig. 4(a), the tilted angle between the slit and groove is 5° and *L* varies gradually and continuously from 1.15 μm to 4.78 μm. The geometric parameters of fabricated slits and grooves are identical to those used in numerical simulation (i.e., *w*_*1*_ = 130 nm, *w*_*2*_ = 230 nm and *d* = 70 nm). As illustrated by Fig. S2(a) in SI, a collimated white light through a tunable liquid-crystal filter (TLCF, Cambridge Research & Instrumentation, VIS-07,) with a ~7 nm bandwidth is employed to illuminate the sample. The transmitted light is collected by a 40X objective lens (Olympus, LUCPLFLN 40X, NA = 0.6) and imaged by a CCD camera (Hamamatsu, C8484-03G02). As shown in Fig. 4(b), clear interference fringes can be observed from the transmission images at λ = 520 nm, 620 nm and 720 nm, from which one dimensional interference fringes versus *L* can be obtained as shown in Fig. 4(c). Within the spectral tunable range of the TLCF from 520 nm to 720 nm, intrinsic phase shift *φ*_*0*_(λ) is extracted from the interference fringes versus *L* as plotted by blue circles in Fig. 4(d). The red dashed line is the polynomial fitting to experimental data. The fluctuation of the extracted phase may be attributed to wavelength tuning precision of liquid crystal filter, fabricated error of nanostructures, metal surface roughness, CCD noise, as well as the inaccuracy in identifying the peak and valley positions of the spatial interference pattern at each wavelength (e.g. see Fig. 4(c)). A more accurate data processing procedure should include multi-image capturing at each wavelength and provide an error bar at each data point, which, however, is more time-consuming. To verify this extracted *φ*_*0*_(λ), we then fabricate three parallel slit-groove structures on the same metal film with constant *L* = 5.3, 6.2, and 7.3 μm, respectively, whose SEM images are shown in Fig. S3 in SI. Other geometric parameters are identical to the tilted one shown in Fig. 4(a). Their transmission spectra are measured by a fiber-coupled spectrometer (see Fig. S2(b) of SI) and normalized using the transmission spectrum of a reference structure (i.e., a single-slit with the same width (*w*_1_ = 130 nm)). As shown by black curves in Fig. 4(e–g), spectral interference fringes can be observed, similar to the modeled curves shown in Fig. 1(b). To compare with these experimental results, analytical interference patterns were plotted using Eq. (2) with a constant *φ*_*0*_, as shown by the blue solid curves. One can see the obvious deviation between blue and black curves. When the extracted dispersive *φ*_*0*_(λ) was substituted into the analytical equation as shown by red dashed curves in Fig. 4(e–g), the plotted interference curves fit very well with the experimental observation, validating the feasibility of the proposed method to extract phase change dispersion.

## Conclusion

In conclusion, we report a practical approach to extract the intrinsic phase change dispersion during the interaction between free-space light, SPP and nanogroove/slit on metallodielectric interfaces. Numerically extracted data agrees very well with results obtained from near-field analysis. The advantage of the proposed method is that only far-field scattering and transmission signals of slit-groove plasmonic interferometer are needed, which is conveniently validated using experiment, and therefore is superior over the previously reported microscopic model requiring near-field information of SPP modes^2^,^22^. Substituting the nanogroove demonstrated in this work with other nanostructures (e.g. nanoslit^4^,^28^, nano-hole^14^, and nano-ridge^40^ etc.), this far-filed interferometric approach is generally feasible to extract the intrinsic phase dispersion associated with SPP generation and scattering, which is particularly important for on-chip quantum plasmonics^41^, magnetic plasmonics^42^,^43^, and sensing and imaging applications^44^,^45^.

## Additional Information

**How to cite this article**: Zeng, X. *et al.* Phase change dispersion of plasmonic nano-objects. *Sci. Rep.* **5**, 12665; doi: 10.1038/srep12665 (2015).

## Supplementary Material

Supplementary Information

## Figures and Tables

**Figure 1:**
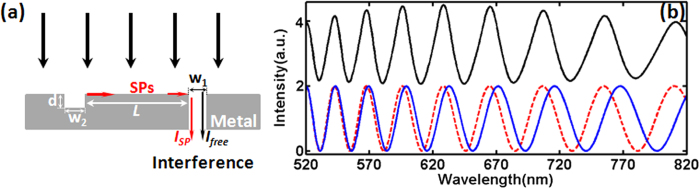
Slit-groove plasmonic interferometer. (**a**) Conceptual illustration of the slit-groove plasmonic interferometer. (**b**) Spectral interference fringes (***L*** = 10 μm) simulated by FDTD method (black) and analytical equation without (blue) or with (red) considering the phase dispersion, *φ*_*0*_(λ).

**Figure 2:**
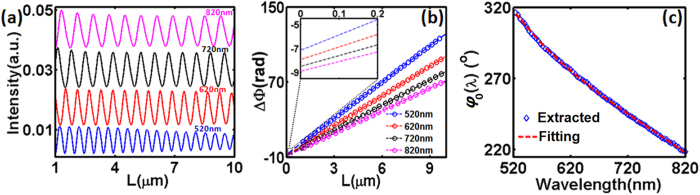
Extraction of the intrinsic phase *φ*_*0*_(λ) from far-field information. (**a**) Interference fringes versus ***L*** at fixed λ of 520, 620, 720 and 820 nm, respectively. **(b)** Empty circles represent the phase ΔΦ of interference peaks and valleys, which are fitted by dashed straight lines. Inset shows the intersection point on the y-axis, i.e. the intrinsic phase, *φ*_*0*_(λ), at *L* = 0. **(c)** Extracted *φ*_*0*_(λ) (blue diamonds) in the spectral region from 520 nm to 820 nm. The red dashed line is the polynomial fitting to extracted data.

**Figure 3:**
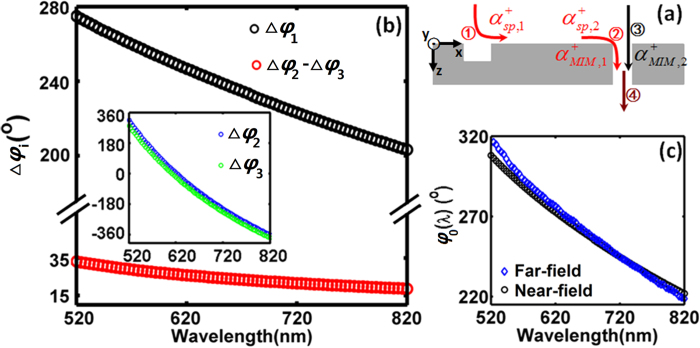
Microscopic understanding of intrinsic phase dispersion. (**a**) Illustration of the scattering processes ① –④ involved in the interaction between two interference components. (**b**) The intrinsic phase dispersion contributed by the scattering processes occurred at the groove and slit, i.e. Δ*φ*_1_ (black) and (Δ*φ*_2_ − Δ*φ*_3_) (red), respectively. Inset: dispersion of Δ*φ*_2_ (blue) and Δ*φ*_3_(green). (**c**) Based on near-field information, intrinsic phase dispersion calculated by Eq. (4) is shown by black circles, which is the summation of black and red circles shown in (**b**). For comparison, the blue diamonds are phase dispersion extracted using proposed far-field approach, which is duplicated from Fig. 2(c).

**Figure 4:**
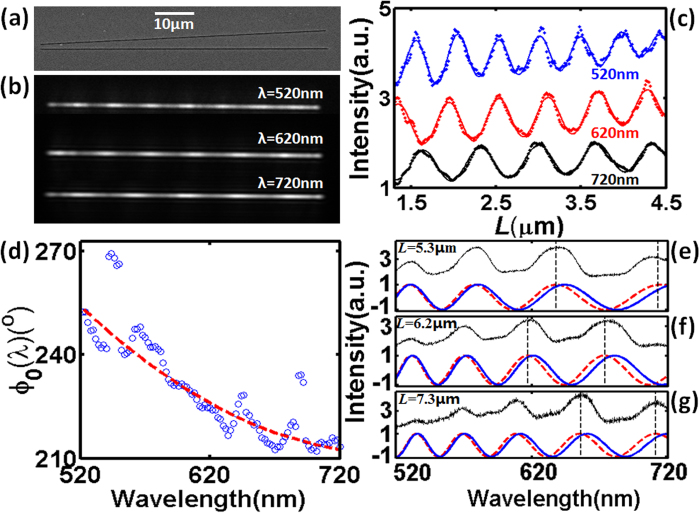
Experimental extraction of phase dispersion from far-field image of tilted slit-groove interferometers. (**a**) SEM image of a tilted slit-groove structure (θ = 5°). (**b**) The transmission image through the tilted slit-groove structure at λ = 520 nm, 620 nm and 720 nm, respectively. (**c**) Measured interference fringes (dots) extracted from (b). Solid curves are fitted curves to identify peak and valley positions. **(d)** Empty circles are intrinsic phase change extracted from the interference fringes in (c), which is fitted by a polynomial function as shown by the red dashed line. (**e**)–(**g**) Spectral interference fringes of parallel slit-groove plasmonic interferometers with (**e**) *L* = 5.3 μm, (f) 6.2 μm, and (**g**) 7.3 μm, respectively. Black curves are measured interference patterns. Red and blue curves are analytical interference patterns with and without considering the phase dispersion obtained in (**d**), respectively.
