# Supplementary material for: Phase change dispersion of plasmonic nano-objects
Source: Sci Rep. 2015 Jul 29;5:12665. doi: 10.1038/srep12665 (PMC4518265; doi:10.1038/srep12665)
Supplement: Supplementary Information [file srep12665-s1.pdf]

Supplementary Information

Phase change dispersion of plasmonic nano-objects

Xie Zeng[1,2], Haifeng Hu[3], Yongkang Gao[4], Dengxin Ji[2], Nan Zhang[2], Haomin Song[2], Kai Liu[2],

Suhua Jiang[1*], Qiaoqiang Gan[2†]

1. *Material Science Department, Fudan University, Shanghai, China 200433*
2. *Department of Electrical Engineering, The State University of New York at Buffalo, Buffalo, NY 14260*
3. *College of Information Science and Engineering, Northeastern University, Shenyang 110819, China*
4. *Alcatel-Lucent Bell Labs, Murray Hill, NJ 07974*

* Email: jiangsh@fudan.edu.cn; † Email: qqgan@buffalo.edu

I. Theoretical modeling using finite difference time domain (FDTD) method

The FDTD modeling was performed using RSoft ® FullWAVE module [S1]. A transverse magnetic (TM) plane wave is launched to illuminate the nanogroove and nanoslit simultaneously. A detector is placed at the other side of the slit to collect the scattered light intensity. A non-uniform gridding is used, where grid sizes in the bulk material are $\Delta x_{bulk} = \Delta z_{bulk} = 20$nm, and grid sizes at structure edges are $\Delta x_{edge} = \Delta z_{edge} = 10$nm. The minimum grid number between two neighboring interfaces is set to 10. Perfectly matched layers (PML) with a 0.5μm width are used to wrap the computation area to eliminate reflection at these boundaries. For the dispersive permittivity of silver $\varepsilon_m(\lambda)$, we used the six-term Lorentz model provided by RSoft ® material library [S1], which fits the experimental data [S2] very well in the spectral regime of interest (see Fig. S1).

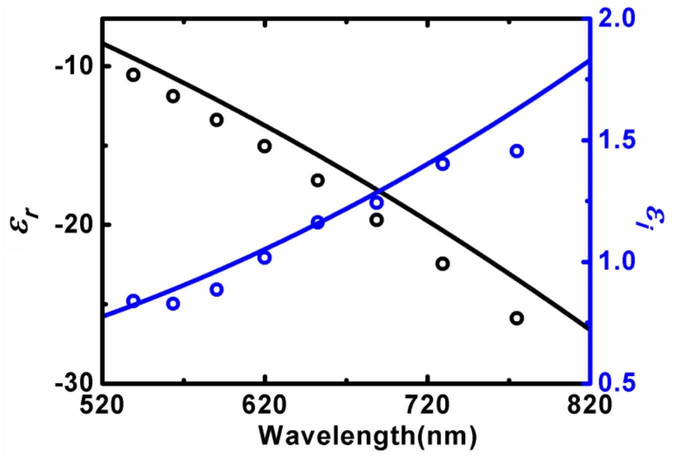

Fig. S1 | Optical constant comparison. Real (black) and imaginary (blue) parts of silver permittivity provided by RSoft ® (lines) [S1] and measured data (circles) [S2].

II. Microscopic modal for calculation of intrinsic phase shift

(i) Phase of SPP modes excited by nanogrooves

When the TM-polarized incident light impinges on the slit-groove structure, the groove will couple free-space light into SPPs propagating towards the slit via the scattering process ① shown in Fig.3 (a). The phase of SPPs can be calculated by a microscopic model proposed by Lalanne *et.al.* [S3]. According to the reciprocity theorem of Maxwell's equations [S4], every two modes in an absorbing waveguide should satisfy the unconjugate general form of orthogonality condition, as described by Eq.(S1),

$$\int_{-\infty}^{+\infty} E_{z,\rho}^{+} H_{y,\rho'}^{-} - H_{y,\rho}^{+} E_{z,\rho'}^{-} dz = 0, \text{ for } \rho \neq \rho' \quad \text{(S1)}$$

Here ρ and ρ′ represent two arbitrary modes in the waveguide. The '+' and '-' signs are used to distinguish the forward-propagating and backward-propagating modes along the x-axis. The total EM field on the Ag/Air interface can be expressed as:

$$E_z(x,z) = \alpha_{sp}^{+} E_{z,sp}^{+}(x,z) + \alpha_{sp}^{-} E_{z,sp}^{-}(x,z) + \sum_{\sigma \neq sp} c_\sigma E_{z,\sigma}(x,z) \quad \text{(S2.a)}$$

$$H_y(x,z) = \alpha_{sp}^{+} H_{y,sp}^{+}(x,z) + \alpha_{sp}^{-} H_{y,sp}^{-}(x,z) + \sum_{\sigma \neq sp} c_\sigma H_{y,\sigma}(x,z) \quad \text{(S2.b)}$$

Here $\{E_{z,sp}, H_{y,sp}\}$ represents the SPP mode propagating on the Ag/Air interface, and $\{E_{z,\sigma}, H_{y,\sigma}\}$(σ≠sp) are radiative modes other than SPP mode. Based on the expansion of the total field $\{E_z, H_y\}$ described in Eq.(S2), one can get

$$\begin{aligned}&\int_{-\infty}^{+\infty} E_z(x,z) H_{y,sp}^{-} - H_y(x,z) E_{z,sp}^{-} dz \\ &= \alpha_{sp}^{+} \int_{-\infty}^{+\infty} (E_{z,sp}^{+} H_{y,sp}^{-} - H_{y,sp}^{+} E_{z,sp}^{-}) dz + \sum_{\sigma \neq sp} c_\sigma \int_{-\infty}^{+\infty} E_{z,\sigma}^{+} H_{y,sp}^{-} - H_{y,\sigma}^{+} E_{z,sp}^{-} dz\end{aligned} \quad \text{(S3)}$$

Due to the orthogonality condition described in Eq. (S1), the last term on the right side of Eq. (S3) is zero. Consequently, complex amplitudes of SPP modes propagating on Ag/Air interface can be calculated using the following equation (i.e. Eq. (3) in the main text),

$$\alpha_{sp}^{\pm}(x) = \pm \frac{\int_{-\infty}^{+\infty} \left[E_z(x,z) H_{y,sp}^{\mp}(z) - H_y(x,z) E_{z,sp}^{\mp}(z) \right] dz}{\int_{-\infty}^{+\infty} \left[E_{z,sp}^{+}(z) H_{y,sp}^{-}(z) - H_{y,sp}^{+}(z) E_{z,sp}^{-}(z) \right] dz} \quad \text{(S4)}$$

Here '+' and '-' signs actually refer to SPP waves propagating rightward and leftward along x-axis in Fig.3(a), whose intensity and phase can be expressed by $|\alpha_{sp}^{\pm}|^2$ and $\arg(\alpha_{sp}^{\pm})$, respectively [S5]. The total field $\{H_y(x,z), E_z(x,z)\}$ can be modeled using full-wave FDTD modeling, while the SPP field $\{H_{y,sp}(z), E_{z,sp}(z)\}$ can be calculated analytically using following equations [S6]:

$$H_{y,sp}(z) = \begin{cases} \exp(\gamma_d z), & z < 0 \\ \exp(-\gamma_m z), & z > 0 \end{cases} \quad \text{(S5.a),}$$

$$E_{z,sp}(z) = \begin{cases} \dfrac{k_{sp}}{\omega \varepsilon_m} \exp(\gamma_d z), & z < 0 \\ \dfrac{k_{sp}}{\omega \varepsilon_d} \exp(-\gamma_m z), & z > 0 \end{cases} \quad \text{(S5.b).}$$

where ω is the angular frequency of the incident light; ε_m and ε_d are permittivities of metal (Ag) and dielectric (Air) materials. The propagation constant of SPP mode is $k_{sp} = (\omega / c)\sqrt{\varepsilon_m \varepsilon_d / (\varepsilon_m + \varepsilon_d)}$, and $\gamma_d = \sqrt{k_{sp}^2 - \varepsilon_d \omega^2}$, $\gamma_m = \sqrt{k_{sp}^2 - \varepsilon_m \omega^2}$.

(ii) Phase of the waveguide mode confined in the metal-insulator-metal (MIM) nanoslit

As shown in Fig.3 (a), the slit couples SPPs and the incident free-space light into the fundamental mode of the MIM waveguide (i.e., the nanoslit), corresponding to scattering processes ② and ③, respectively. Using the method described above, the complex amplitudes of MIM fundamental modes, $\alpha_{MIM}^{\pm}$, can also be calculated by properly modifying the subscripts and coordinates of Eq. (S4),

$$\alpha_{MIM}^{\pm}(z) = \pm \frac{\int_{-\infty}^{+\infty}\left[E_x(x,z)H_{y,MIM}^{\mp}(x) - H_y(x,z)E_{x,MIM}^{\mp}(x)\right]dx}{\int_{-\infty}^{+\infty}\left[E_{x,MIM}^{+}(x)H_{y,MIM}^{-}(x) - H_{y,MIM}^{+}(x)E_{x,MIM}^{-}(x)\right]dx} \quad \text{(S6)}$$

Here '+' and '-' represent MIM fundamental mode propagating downwards and upwards along the slit along z axis in Fig. 3(a). Similarly, their phase can be calculated by $\arg(\alpha_{MIM}^{\pm})$. The field distribution of MIM fundamental mode $\{H_{y,MIM}(z), E_{x,MIM}(x)\}$ can be expressed by following equations [S7]:

$$H_{y,MIM}(x) = \begin{cases} \exp\left[\gamma_m(x + w/2)\right], & x < -w/2 \\ A\exp\left[\gamma_d(x + w/2)\right] + B\exp\left[-\gamma_d(x + w/2)\right], & -w/2 < x < w/2 \\ C\exp\left[-\gamma_m(x - w/2)\right], & x > w/2 \end{cases} \quad \text{(S7.a)}$$

$$E_{x,MIM}(x) = \begin{cases} \frac{\beta}{k_\omega \varepsilon_m}\exp\left[\gamma_m(x + w/2)\right], & x < -\frac{w}{2} \\ \frac{A\beta}{k_\omega \varepsilon_d}\exp\left[\gamma_d(x + w/2)\right] + \frac{B\beta}{k_\omega \varepsilon_d}\exp\left[-\gamma_d(x + w/2)\right], & -w/2 < x < w/2 \\ \frac{C\beta}{k_\omega \varepsilon_m}\exp\left[-\gamma_m(x - w/2)\right], & x > \frac{w}{2} \end{cases} \quad \text{(S7.b)}$$

where w is the width of the MIM waveguide (i.e. the slit width w_1); β is the propagation constant of MIM fundamental mode, which can be calculated by solving the eigenvalue equation of the MIM waveguide; $k_\omega = \omega/c$ is the wave vector in the vacuum; $\gamma_d = \sqrt{k_\omega^2 \varepsilon_d - \beta^2}$ and $\gamma_m = \sqrt{\beta^2 - k_\omega^2 \varepsilon_m}$. The coefficients, A, B and C, can be obtained by matching the boundary conditions at the two interfaces of the MIM waveguide (i.e. at $x=-w/2$ and $x=w/2$).

(iii) Calculation of the intrinsic phase shift

In Fig. 3(a), $\alpha_{sp,1}^{+}$ and $\alpha_{sp,2}^{+}$ represent the complex amplitudes of forward-propagating SPP generated at the groove and arriving at the slit, respectively; $\alpha_{MIM,1}^{+}$ and $\alpha_{MIM,2}^{+}$ are complex amplitudes of MIM fundamental modes coupled from SPP and free-space light, respectively.

According to Eq. (4) and (5) in the main text, the intrinsic phase shift can be calculated at each wavelength by the following equation:

$$\varphi_0 = \arg(\alpha_{sp,1}^{+}) - arg(\alpha_{sp,2}^{+}) + arg(\alpha_{MIM,1}^{+}) - arg(\alpha_{MIM,2}^{+}) \qquad \text{(S8)}$$

where all parameters can be calculated using Eq. (S4)-Eq. (S7) described above.

III. Experimental details

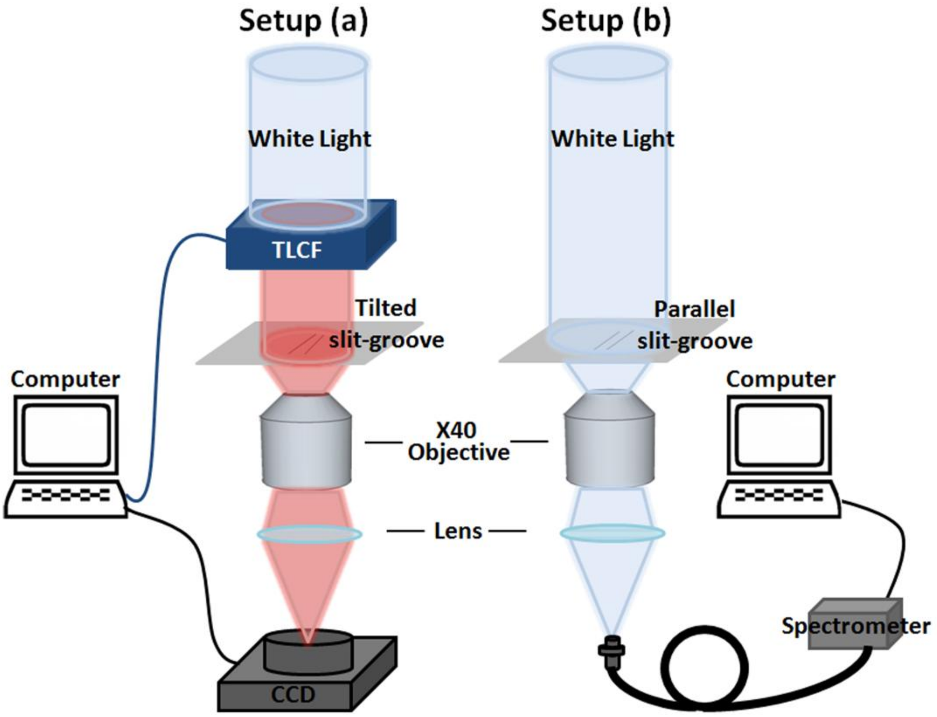

Fig.S2 | Schematics of experimental setup. Systems shown in (a) and (b) are used to mesuare tilted and parallel slit-groove plasmonic interferometers, respectively. TLCF: Tunable Liquid-Crystal Filter. CCD: Charge-Coupled Device

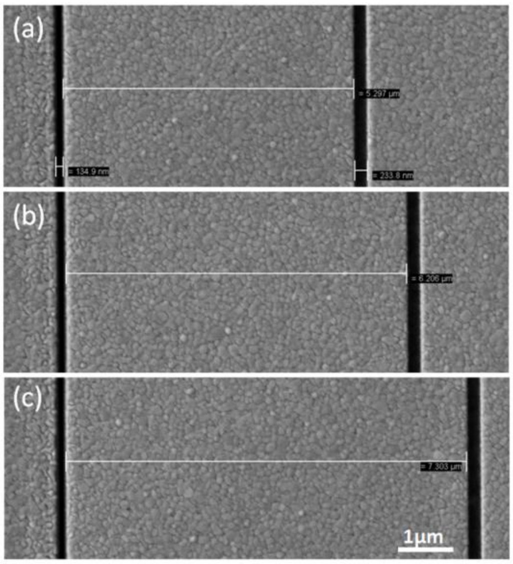

Fig.S3 | **SEM images of parallel slit-groove plasmonic interferometers. (a)** *L*= 5.3 µm, **(b)** *L*= 6.2 µm, and **(c)** *L*= 7.3 µm.

References

[S1] http://optics.synopsys.com/rsoft/

[S2] Palik, E. D. *Handbook of Optical Constants of Solids* (Academic Press, 1998).

[S3] Lalanne, P., Hugonin, J. P. & Rodier, J. C. Approximate model for surface-plasmon generation at slit apertures. *J. Opt. Soc. Am. A* **23**, 1608-1615 (2006).

[S4] Snyder, A. W. & Love, J. D. *Optical waveguide theory*. (Chapman and Hall, 1983).

[S5] Lalanne, P., Hugonin, J. P., Liu, H. T. & Wang, B. A microscopic view of the electromagnetic properties of sub- metallic surfaces. *Surf. Sci. Rep.* **64**, 453-469 (2009).

[S6] Raether, H. *Surface Plasmons on Smooth and Rough Surfaces and on Gratings*. (Springer, 1988).

[S7] Hu, H. *et al.* Surface plasmon coupling efficiency from nanoslit apertures to metal-insulator-metal waveguides. *Appl. Phys. Lett.* **101**, 121112-121115 (2012).
